# Knowledge and adherence to antiretroviral therapy among adult people living with HIV/AIDS treated in the health care centers of the association "Espoir Vie Togo" in Togo, West Africa

**DOI:** 10.1186/1472-6904-10-11

**Published:** 2010-09-17

**Authors:** Yao Potchoo, Kpatcha Tchamdja, Agnon Balogou, Vincent P Pitche, Innocent P Guissou, Etienne K Kassang

**Affiliations:** 1 Université de Lomé, Faculté Mixte de Médecine et de Pharmacie, BP 1515, Lomé - Togo; 2 Centre Hospitalier Universitaire de Kara, Service de Pharmacie BP 18 - Kara - Togo; 3 Université de Ouagadougou - UFR/SDS 03 BP 7021 Ouagadougou 03 - Burkina Faso; 4 Espoir Vie Togo, Région Centrale (EVT/RC) - Togo

## Abstract

**Background:**

The efficiency of antiretroviral therapy (ART) depends on a near perfect level of patients' adherence. The level of adherence of adults HIV-infected patients treated in the HIV/AIDS health care centres of the association "Espoir Vie Togo" in Togo, West Africa is not properly documented. The aim of the present study was to examine by means of self-reports the knowledge, the adherence level and associated factors to antiretroviral therapy (ART) among these patients.

**Methods:**

We conducted a cross-sectional survey among adult people living with HIV/AIDS (PLWHA) through a structured questionnaire.

**Results:**

A total of 99 patients were enrolled. Among them, 55.6% knew the name of antiretroviral agents of regimens prescribed. All patients had a good knowledge of treatment schedule. The treatment regimens based on 2 NRTIs + 1 NNRTI were used in 90% of patients. The average adherence rate was 89.8% of the total doses prescribed while 62.62% of patients showed an adherence rate of 95% or above. The treated groups were similar in term of median % of medication doses taken according to PLWHA epidemiological characteristics. However, patients reported forgetting (34.9%), travel (25.6%), cost of treatment (13.9%) and side effects (11.6%) as the main factors of missing at least once a dose intake.

**Conclusion:**

These results should encourage the association and all the involved actors in the HIV/AIDS's program to strengthen counseling, education and information interventions for HIV-infected patients in order to overcome the potential barriers of poor adherence.

## Background

Since 1996, progress in the field of antiretroviral therapy has led to the reduction of about 80% of deaths, the number of cases of acquired immunodeficiency syndrome (AIDS) and the incidence of opportunistic infections [1]. About twenty drugs belonging to 4 classes defined according to their pharmacological modes of action constitute the current arsenal of antiretroviral drugs (ARVs). The combinations of these drugs have dramatically changed the prognosis of an infection which natural consequence is death for over 90% of the patients into chronic infection [2,3]. The long-term nature of the disease has further complicated its management. In this context, sustained adherence is an essential tool of the long-term efficiency of ARVs therapy [4] (e.g. significant reduction in viral load, drug resistance, deterioration of health status and treatment failure) [5-8]. In addition, the role of the knowledge of treatment regimens [9] and cognitive demands related to the complexity of ARVs pharmacotherapy [10,11] were reported as factors that may influence the level of adherence [12,13]. Recent studies on patients' adherence in African health contexts showed that it is relatively higher than that of industrialized countries health context [14,15].

Cohorts followed up in developed countries have emphasized the frequency of non-adherence situations, the high level of adherence required to obtain optimal efficiency of ARVs chemotherapy, and delay the beginning of the human immunodeficiency virus (HIV) resistance and pejorative evolution of the disease [6,16].

The assessment of adherence, parameter that has no standard method of measurement, which varies over time, meets a number of methodological difficulties [5]. Few studies have documented the factors involved and the level of adherence of adults people living with HIV/AIDS (PLWHA) to ARVs therapy in Togo, West Africa. The objectives of the present study were to assess the knowledge of the treatment regimens prescribed to patients; quantify the level of adherence to ARVs therapy and identify the factors related to non-adherence.

### Eligible patients and method

The present study was a cross-sectional survey carried out during the months of August and October 2005 in the HIV/AIDS health care centers (in urban areas of Lomé and Sokodé) of the association "Espoir Vie Togo" (EVT), the first organized non-governmental organization of people living with HIV/AIDS and those contributing to their support. The association is recognized by National HIV/AIDS Control Programme "Programme National de Lutte contre le SIDA" (PNLS-Togo) as an ambulatory HIV/AIDS health care association. The association is also involved in the purchasing of ARVs for its members from the "Centrale d'Achat de Médicaments Essentiels et Génériques" (CAMEG-Togo). The ARVs are supplied to the patients, in EVT's pharmaceutical stores.

We interviewed eligible HIV-infected adults when they came to EVT's pharmaceutical stores to receive their monthly supply of ARVs. The enrolment was done on a voluntary basis.

### Inclusion criteria

To be enrolled in the study, HIV-infected patients have to meet the following criteria: all adult patients over 15 years, the members of EVT association placed on ARVs therapy for over 1 month, who receive ARVs in EVT pharmaceutical stores. Hospitalized patients and those whose age is below 15 years were excluded from the study.

### Data collection method and variable of interest

A cross-sectional survey was conducted from a structured questionnaire (additional file 1) submitted to enrolled patients (face-to-face interviews for about 30-45 mn). The relevant data were collected for a period covering one month per EVT's health care centre (August and October 2005). Data collected concerned the following variables:

- The characteristics of patients (sex, age, educational level) and ARVs procurement;

- The patient treatment knowledge which was assessed through the knowledge of the names of the various drugs prescribed their respective dosages (number of tablets per dose and number of daily intakes) and the times of intake. The statements of patients were compared with the prescriptions and the treatment regimens mentioned in their health card;

-The type of ARVs combination regimens prescribed;

- The rate of adherence to treatment by means of self-reporting: It was assessed in the last month and defined as the number of doses taken during the last 7 days before each interview. Adherence score was expressed as the proportion (%) of tablets taken compared to prescribed tablets. An adherence rate of 95% or more was considered to be good.

-The factors that induced poor adherence to treatment.

Given that the Ethic Committee was not available at the period of the study, the Ministry of the Health authorized the survey.

### Statistical analyses

The median % of medication doses taken by treated groups were compared using ANOVA method (Sigma Stat32 software; Jandal Corp, San Rafael, CA): t test was used for the sex factor; Mann-Whitney Rank sum test was used for the ARVs procurement and the ARVs combination prescribed and Kruskal-Wallis one-way analysis of variance on ranks method was used for the age groups and the educational level. The difference between the groups was considered to be significant if p < 0.05.

## Results

A total of 99 patients (77.3%) of 128 interviewed during the investigation, accepted to be submitted to the questionnaire.

### Sex and age of patients

Of the total sample (n = 99), 76 were female (76.8%) and 23 male (23.2%) with a sex ratio of 0.3. The age of patients ranged between 21 to 57 years, with an average age of 36.8 years. The age group from 36 to 45 years was more representative (39.4%), followed by 26 to 35 years (34.3%).

### ARVs procurement

Among the patients included in this study, sixty one percent were under an individual financial participation of "5 000 fcfa" per month (approximately US $ 11 reduced to US $ 2 since November 2005), the remaining received ARVs free of charge.

### Treatment regimen knowledge

Forty four patients (44.4%), knew perfectly the names of various ARVs included in the combination regimens. All respondents had a perfect knowledge of the treatment schedule (number of tablets per dose, the number of daily intakes and the times of drug intake).

### Types of ARVs combinations prescribed

All patients have received a combination of three ARVs. Four different ARVs combinations (ARVs-GF (Global Fund)) were legalized, of which one available fixed-dose combination (lamivudine + stavudine + nevirapine). Only 39.4% of patients have received this fixed-dose combination as against 60.6% who were placed on non-fixed-dose combinations.

**Figure 1:**
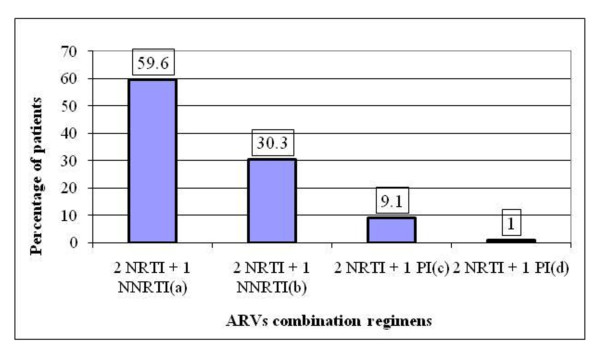
**Distribution of patients according to ARVs combination regimens prescribed**. (a): 3TC+D4T+NVP; (b): AZT+3TC+EFV; (c): ddi+D4T+IDV; (d): ddi+D4T+NFV; AZT = zidovudine; 3TC = lamivudine; EFV = efavirenz; D4T = stavudine; NVP = nevirapine; ddi = didanosine; IDV = indinavir; NFV = nelfinavir.

The treatment regimen containing 2 nucleoside reverse transcriptase inhibitors (NRTIs) + 1 non-nucleoside reverse transcriptase inhibitor (NNRTI) was used in 89.9% of patients; While the remaining 10.1% were placed on 2 NRTIs + 1 protease inhibitor (PI) based regimens (Figure 1).

### Adherence rate to ARVs therapy

The average individual adherence level was 89.8% of doses taken while sixty two (62) patients (62.62%) reported 95% or more of the doses taken. Table 1 shows the distribution of median % of medication doses taken according to the PLWHA epidemiological characteristics and P values. Except patients group who received 2 NRTIs + 1 PI combination (median percent of 46% of doses taken), the median percent of other treated groups varied from 82% to 92% of doses prescribed.

**Table 1:** Distribution of patients and median percent of medication doses taken according to the epidemiological characteristics

Epidemiological characteristics		n	Median % of doses taken	p
Sex	Female	28	85.00	0.604^a^
	Male	9	92.00	
				
Age groups	15-25 years	3	85.00	0.838^b^
	26-35 years	14	88.50	
	36-45 years	11	85.00	
	>45 years	9	92.00	
				
Educational level	Non scolarised	8	82.00	0.536^b^
	Primary level	12	92.00	
	Secondary level	16	89.00	
	Universitary level	1	85.00	
				
Triple ARVs therapy received	2 NRTIs + 1 NNRTI	35	85.00	0.638^c^
	2 NRTIs + 1 PI	2	46.00	
				
	Fixed combination	17	85.00	0.626^c^
	Non fixed combination	20	87.00	
				
ARVs procurement	Offered ARVs free of charge	16	85.00	0.269^c^
	Fixed participation of 11$/month	21	92.00	

n: number of patients; a: t-test; b: Kruskal-Wallis one way ANOVA on ranks; c: Mann-Whitney Rank Sum Test

### Factors of non-adherence

**Figure 2:**
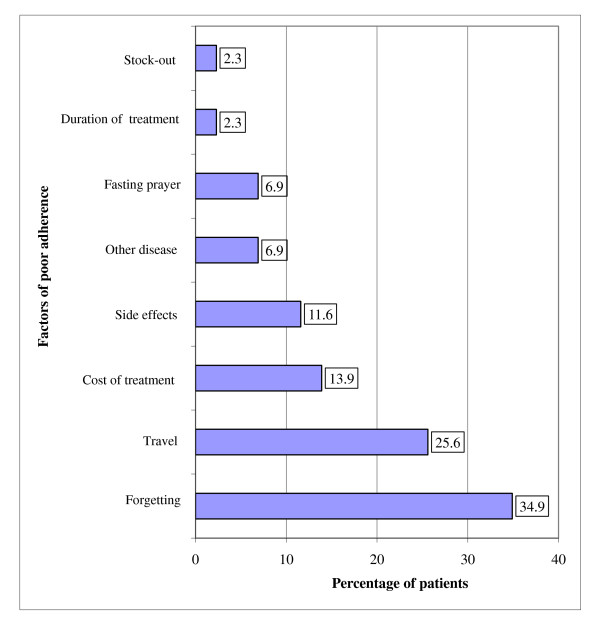
**Distribution of patients (n = 43) according to the factors of poor adherence**.

Forgetting (34.9%), travel (25.6%), cost of treatment (13.9%) and side effects (11.6%) were the main factors of poor adherence reported by the 43 patients who missed at least once a dose intake (Figure 2). They reported using a method in order not to forget the medication intake. Among them 69.4% stated using a watch and/or an alarm clock to remember the time of drug intake, while 27.9% referred to parents' recall for the medication intake.

Twelve patients (12.1%) stopped or changed one or more ARVs included in the treatment regimens because of side effects in 6 patients (6.1%), inefficiency in 4 patients (4%) and because of disease in 2 patients (2%).

## Discussion

The assessment of the adherence level by interviewing PLWHA seems to be an acceptable method in Africa. It is simple, cheap and accessible. This method has, however some limits (the length of interview, the subjectivity of patient statements). The fact that the investigator is unknown to the patients, that he is not a member of the health care team and the guarantee of patient's anonymity limit this bias (e.g. the patients were not afraid of being criticized for poor adherence). An additional objective method (e.g. a count of returned pills) would have helped to improve the estimation of adherence level.

The enrollment of members of the association could lead to a biased selection if the survey acceptation is related to a certain profile of patients that we didn't clarified. However, this bias was limited because the enrollment concerned consecutive patients attending EVT health care centres during the period of the study.

The sex ratio in the present study (0.3 with 76.8% of women) is twice as high compared to that observed by PNLS [17] and lower than that of South Africa (0.4) [18], Uganda (0.5) [19] and Senegal (1) [20] cohorts. Previous reports in Senegal [21], Morocco [22] and Benin [23] have showed the highest proportion of male. The patients' average age is similar to that of PNLS [17] but less than that reported in Senegal (38 years) [20] and higher than that observed in Kenya (31.5 years) [24], Morocco (35.5 years) [22] and in Bothswana (35.6 years) [25].

The whole eligible patients followed the regimens and the treatment schedule prescribed compared to Diabate et al's report in Ivory Cost (76.2% of the patients followed their regimen special instructions as against 63.6% who took their medication at the prescribed time) [26]. However, only 55.6% (n = 55) knew perfectly the names of ARVs prescribed. This proportion can be explained by the high proportion of illiterate patients (19.2%). As previously reported, treatment knowledge [9] and the cognitive demands related to complexity of ARVs pharmacotherapy [10,11] have been targeted as necessary components of adherence.

Standard triple-ARVs combination regimens were prescribed. Four types of ARVs combinations of which two were based on 2 NRTIs + 1 NNRTI (standard first-line regimen proposed by WHO) and two on 2 NRTIs + 1 PI (Figure 1) were used compared to 16 different combinations reported by Roux et al [23] in Benin.

About 90% (n = 89) of patients were in 2 NRTIs + 1 NNRTI-based regimens (Figure 1). This proportion is similar to that observed by PNLS-Togo (90.8%) after a national evaluation of ARVs therapy [17]. These regimens available in many other sub-Saharan African countries [18-20,25,26] are recommended because of their virological and immunological efficacy. They contribute to improve adherence as there is an available fixed-dose combination (lamuvidine + stavudine + nevirapine) as first-line ARVs regimen. This fixed-dose combination available in other African countries [19,26] used by 39.4% (n = 39) of patients was highly appreciated because it requires only 2 daily intakes of one tablet, promoting adherence. However, in this study, we didn't observe any statistical difference between treated groups placed on a fixed-dose combination and treated groups who received non-fixed-dose combinations.

The average adherence rate was close to that observed in Senegal (91%) [21], but lower than that reported by Vriesendorp et al [25] in Botswana (98%) on the basis of patients' reports. Several studies showed that 90% to 95% of medication doses should be taken for optimal virologic suppression and reduction of virologic failure [27] but according to two studies, a moderate adherence (80-90%) should lead to a better viral suppression under more potent regimen including NNRTI [28,29]. Otherwise, adherence rate of 95% or greater is strongly correlated with CD4 cell count increase, viral load and morbidity decreases [6]. The proportion of patients with adherence level of 95% or more (62.62%) is less compared to previous reports in Ivory Cost (74.3%) [26]. Adherence level could be improved if adherence data sheets updated by prescribers were available allowing patients follow-up.

Table 1 includes median % of medication doses taken according to patients' epidemiological characteristics. No significant difference have been observed between the treated groups according to the sex, the age groups, the educational level, the ARVs regimen prescribed and the ARVs procurement (p > 0.05). Ours findings confirm those of previous studies [19,30] concerning these three first factors (excluding younger age). This lack of difference among treated groups could be explained by the sensitization of the PLWHA on the role of ARVs therapy adherence by the association ''Espoir Vie Togo''.

The existence of tools or methods to promote the adherence was also reported. In the present study, 43.4% (n = 43) of patients reported using at least one (69.4% recognized using a watch/alarm clock to remember the times of medication intake) compared to 27.9% who stated being reminded by parents.

Forgetting, travel, cost of treatment and side effects (Figure 2) have been cited by 43 patients having missed at least one dose of treatment as the main factors influencing the adherence score and often reported in similar studies [15,19,24]. Missing drug intake is mainly related to a state of well being feeling experienced by the patients, lack of a square meal a day (specific factor in Africa) [31-33] on which the medication intake is regulated and a feeling of weariness suitable for all long-term treatment. There is therefore the need to strengthen advice and information on the consequences of poor adherence for the patient himself and the threat for the public health when he does not take his medications.

Travels were the second main factor of poor adherence. This factor of non-adherence reveals the problem of the lack of a pill container containing at least all daily doses.

The cost of treatment comprising transport, food support and laboratory tests were cited as main obstacles to optimal adherence. Previous studies have reported the incidence of payment factor on the rate of loss in HIV-infected patients' follow-up and/or adherence in Kenya [24]. The risk of reduction in terms of loss to follow-up related to offering ARVs free of charge was 56.6% in cohorts study [24].

Side effects were the 4th barrier of non adherence (11.6%). They were collected on the basis of patients' reports. As a result, we have not considered appropriate to mention them since they are not validated as such in contrast to the report of Bhengu et al [34] who conducted a study on the prevalence of symptoms reported by patients. They have been also reported (including their severity) as main factors for poor adherence in previous studies [9,25,35,36]. On the contrary, Bhengu et al [34] observed no significant relationships between adherence and the intensity of symptoms. Of the 99 patients included in this study, 89 patients (89.9%) reported side effects due to ARVs treatment received. This proportion is higher than that reported in a national survey by PNLS (31.6% of PLWHA) [17]. In the present study, five patients (11.6%, n = 43) failed to take their medication because of side effects. The influence of these side effects may be lessened by a good patient information and training to enable them to handle themselves some minor side effects.

A total of 12 patients (12.1%) stopped or changed one or more ARVs of the combination regimen for side effects, inefficiency of treatment and for illness conditions compared to 49.9% reported in the national survey conducted by PNLS [17].

Overall, a review of the literature shows that the ART-adherence determinants include institutional, socio-economical, psychological and therapeutic factors to take in account in the management of PLWHA care. However, the present study didn't observe any epidemiological characteristic associated with poor adherence (medication doses taken less than 95%). To reach optimal adherence level (100%), our findings suggest the improvement of PLWHA follow-up with the help of adherence data sheets, the counseling/information/education interventions concerning the virological, biological, therapeutic and public health risks of non-adherence to ARVs therapy.

## Conclusion

Our results have shown that the knowledge and the capacity for adherence to ARVs treatment of PLWHA in the present study are satisfactory but have not reached the optimum desirable level. However, these results reflect the quality of the association "Espoir Vie Togo" interventions toward PLWHA. They should encourage the association EVT and all actors involved in the fight against AIDS to maintain and strengthen counseling, education, training and information interventions for PLWHA with a view to overcoming the potential barriers of poor adherence, given that non-adherence leading to the development of ARVs-resistant HIV is a public health concern.

## Abbreviations

AIDS: Acquired Immune Deficiency Syndrome; ART: Antiretroviral Therapy; ARVs: Antiretrovirals; ARVs-GF: Antiretrovirals supported by Global Fund; CAMEG-Togo: Centrale d'Achat de Médicaments Essentiels et Génériques; EVT: Espoir Vie Togo (Association); HIV/AIDS: Human Immunodeficiency Virus/Acquired Immune Deficiency Syndrome; HIV: Human Immunodeficiency Virus; NNRTIs: Non-Nucleoside Reverse Transcriptase Inhibitors; NRTIs: Nucleoside Reverse Transcriptase Inhibitors; PI: Protease Inhibitor; PLWHA: People Living with HIV/AIDS; PNLS-Togo: Programme National de Lutte contre le SIDA; WHO: Word Health Organization.

## Competing interests

The authors declare that they have no financial or no non-financial competing interests in relation to the present manuscript.

## Authors' contributions

YP conceived, designed and coordinated the study, he carried out statistical analysis and drafted the manuscript; KT participated in the design of the study and the questionnaire, he carried out the study (interviewing and data collection), used the data collected for sustaining a doctor thesis of pharmacy; AB, VPP and IPG contributed by means of their respective competence and their experiences to the manuscript reviewing critically for its intellectual content. All authors read and approved de final version of the manuscript; EKK facilitated the interview and offered some facilities to the interviewer.

All authors read and approved the final manuscript.

## Pre-publication history

The pre-publication history for this paper can be accessed here:

http://www.biomedcentral.com/1472-6904/10/11/prepub

## Supplementary Material

Additional file 1 **Questionnaire for assessing PLWHA's knowledge and adherence level to antiretroviral therapy**. The questionnaire sought the epidemiological characteristics of PLWHA, their knowledge of ARVs treatment, their adherence level to ART, the factors of poor adherence, the side effects reported and the prescription of treatment. Click here for file
