# Supplementary material for: Knowledge and adherence to antiretroviral therapy among adult people living with HIV/AIDS treated in the health care centers of the association "Espoir Vie Togo" in Togo, West Africa
Source: BMC Clin Pharmacol. 2010 Sep 17;10:11. doi: 10.1186/1472-6904-10-11 (PMC2949664; doi:10.1186/1472-6904-10-11)
Supplement: Additional file 1 — Questionnaire for assessing PLWHA's knowledge and adherence level to antiretroviral therapy. The questionnaire sought the epidemiological characteristics of PLWHA, their knowledge of ARVs treatment, their adherence level to ART, the factors of poor adherence, the side effects reported and the prescription of treatment. [file 1472-6904-10-11-S1.DOC]

**Additional files**

**Questionnaire for assessing PLWHA’s knowledge and adherence level to antiretroviral therapy**. The questionnaire sought the epidemiological characteristics of PLWHA, their knowledge of ARVs treatment, their adherence level to ART, the factors of poor adherence, the side effects reported and the prescription of treatment.

**THE INVESTIGATION FORM ABOUT THE ADHERENCE OF THE ARVs TREATMENT**

FORM N° /___/___/___/

**I. PATIENT**

1. AGE /___/___/ Years old

2. SEX /___ / 1= male 2= female

3. LEVEL OF STUDY /___/

1= non scholarised 2= Primary 3=Secondary 4=University

4. PROFESSION /___/

1= without employment 2=Salaried (private/public sector) 3= Retired 4= farmer

5= artisan 6=trader 7=student/pupil 8= apprentice

5. MONTHLY INCOME /___/___/___/___/___/___/___/___/ f cfa

6. WHEN HAVE YOU BEEN DEPISTED AS POSITIVE FOR HIV?

Month /___/___/ Year /___/___/___/___/

7. WHEN DO YOU START TAKING THE ARV?

Month /___/___/ Year /___/___/___/___/

8. ARE YOU SUPPORTED IN OBTAINING YOUR MEDICINES?

1= Yes 2=No

9. IF YES? WHAT IS YOUR CONTRIBUTION? /___/___/

1= free of charge 2=fixed price (11$)

**II. KNOWLEDGE OF THE TREATMENT**

10. DO YOU KNOW ARV’s MEDICINE PRESCRIBED TO YOU? /___/

1=Yes 2=No

11. KIND OF ARVs-FM TREATMENT /___/

T1 = 3TC+D4T+NVP T2 = AZT+3TC+EFV T3 = ddi+D4T+IDV  T4 = ddi+D4T+NFV

12. IS IT A COMBINATION TREATMENT? /___/

1=Yes 2=No

13. WHAT KIND OF THERAPY? (………………here be answered by the investigator) / /

1= single ARV regimen 2 = two ARVs regimen 3 = three ARVs regimen

14. WOULD YOU GIVE ME SOME INFORMATION ABOUT THE MEDICAMENT YOU ARE TAKING?

| MEDICAMENTS | NUMBER OF TABLETS  BY TAKING | NUMBER OF  TAKING BY DAY | TAKING  ACCORDING  TO THE MEAL | HOW DRINK WATER |
| --- | --- | --- | --- | --- |
|  | /___/ | /___/ | /___/ | /___/  1= little  2= much |
|  | /___/ | /___/ | /___/ | /___/  1= little  2= much |
|  | /___/ | /___/ | /___/ | /___/  1= little  2= much |

REMARK: TAKING ACCORDING TO THE MEAL:

1=Before 2=During 3=After 4= No particular recommendation

15. HAVE YOU AN OWN METHOD TO REMEMBER TIME AND RECOMMENDATION TO TAKE YOUR MEDICINE? /___/

1= Yes 2=No

16. IF YES, WHICH ONE? /___/

1= Watch 2=one of my family’s member 3=moving with the daily dose

**III. OBSERVANCE OF THE TREATMENT**

17. DURING THE LAST SEVEN (07) DAYS HAVE YOU MISSED TO TAKE YOUR MEDICAMENT? /___/

1=Yes 2=No

18. HOW MANY TIMES? /___/ time(s)

19. HOW MANY TABLETS ARE NOT TAKEN? /___/ tablets

(Count made by the investigator)

20. HAVE YOU GET AT LEAST ONCE SOME INFORMATIONS (ADVISES) ABOUT THE CONSEQUENCES OF A BAD USING OF YOUR TREATMENT? /___/

21. IF YES, FROM WHOM? /___/

1=EVT consultant/ (Adviser) 2= Doctor 3= Parents 4=Friends

**IV. FACTORS OF POOR ADHERENCE**

22. WHAT IS/ARE THE REASON (S) WHY YOU HAVEN’T TAKEN YOUR MEDICINE? /___/

1=forget 2= so long treatment 3=side effects 4=cost of the treatment

5=trip/travel 6=taking times 7= number of tablets to be taken

8=lack of intimacy 9=sickness 10=church 11=feeling be recovered

**V. SIDE EFFETS**

23. HAVE YOU ANY UNDESIRABLE OR SIDES EFFECTS DURING YOUR TREATMENT?

1=Yes 2=No

24. WHICH ONE?

(The investigator explain if necessary the different propositions) /___/____/

1=Asthenia 2=digestive problem 3=abdominal pains 4=swarming 5=headaches 6=shivering of extremities 7=dizziness 8=insomnia

9=itching 10=spots on the skin 11=swollen face 12=cramps

13=joint pains 14=numbing of the legs 15=blackness of nails and palms

16=palpitations 17=constipation 18=appetite loose 19=nightmares

25. HAVE YOU EVER STOPPED OR CHANGED THE MEDICAMENTS (MOLECULES)? /___ /

1=Yes 2=No

26. IF YES, WHY? /___/

1=inefficiency treatment 2=sides effects 3=financial difficulty 4=personal stopped of treatment

**VI. PRESCRIPTION OF THE TREATMENT**

27. ARE YOU SATISFIED WITH YOUR TREATMENT? /___/

1=Yes 2=No

28. WHY? /___/

………………………………………………………………………………………………………………………………………………………………………………………………………………………………………………………………………………………………………………………………………………………………………………………………………………………………………………………………………….............................................
